# Bibliometric analysis of scientific production on university social responsibility in Latin America and the Caribbean

**DOI:** 10.12688/f1000research.141987.1

**Published:** 2023-10-16

**Authors:** Diego Urrunaga-Pastor, Guido Bendezu-Quispe, Deici Dávila-Altamirano, Milagritos N. Asmat, Jordi Grau-Monge

**Affiliations:** 1 Unidad de Responsabilidad Social Universitaria, Facultad de Ciencias de la Salud, Universidad Cientifica del Sur, Lima, Peru; 2 Grupo de Bibliometría, Evaluación de Evidencia y Revisiones Sistemáticas (BEERS), Universidad Cientifica del Sur, Lima, Peru; 3 Centro de Investigación Epidemiológica en Salud Global, Universidad Privada Norbert Wiener, Lima, Peru

**Keywords:** University social responsibility, Latin America, University, Higher education, Community

## Abstract

**Objective:** To evaluate the scientific production on university social responsibility (USR) from institutions in Latin America and the Caribbean.

**Methods:** A bibliometric analysis was conducted on documents published in indexed journals in the Scopus database from its inception until April 2023. Eligible documents included those on USR describing experiences carried out by universities in Latin America and the Caribbean. The number of articles per author, average authors per article, average citations per article, and the number of documents with one or more author were described. Bibliometric indicators regarding authors per article, co-authors per article, and institutional collaboration were presented. Bibliometric networks were constructed based on bibliographic coupling analysis of documents by countries and term co-occurrence in titles and abstracts.

**Results:** Of a total of 4075 documents retrieved from Scopus, 150 were included. Documents published between 1997 and 2023 were identified, with an average annual growth rate of 2.7%. A total of 439 authors were identified, 18 articles had a single author, and an average of 0.3 articles per author and a co-authorship index of 3.13 were found. The percentage of international collaborations was 30.7%. Brazil had the highest proportion of publications (26.4%), followed by Chile (17%) and Colombia (13.2%). *Opción* and *Revista de Ciencias Sociales* were the journals with the highest number of articles published (13 each). In the analysis of term co-occurrence, recent years showed an increase in the use of terms related to e-learning, information and communication technologies, virtual education, COVID-19, sustainable development goals, and URSULA (initiative on USR in institutions in Latin America and the Caribbean).

**Conclusions:** A growth in scientific production on USR in Latin America and the Caribbean was identified. The interest in USR documents in recent years has been focused on COVID-19 and the challenges of virtual education and sustainable development.

## Introduction

In recent decades university social responsibility (USR) has gained increasing importance both in the academic and social spheres. ^1^ USR refers to the commitment of higher education institutions to actively contribute to the sustainable development of society through the generation and application of knowledge, as well as the training of ethical and responsible professionals. ^1^^–^^4^ It is essential to study USR to understand the impact of universities on their environment and foster greater integration between academia and society. ^5^ Research on USR provides valuable insights into practices, challenges, and opportunities related to social responsibility in the university context. ^6^

The scientific literature has addressed USR from different perspectives, but it is necessary to specifically analyze the scientific production in Latin American universities. Several previous studies have analyzed the topic of USR in different regions of the world, such as Europe and globally. ^7^^–^^12^ However, research focused on Latin America and the Caribbean still presents significant gaps in terms of scientific production in this field. Understanding the current situation and trends in USR in Latin American universities will help identify areas for improvement and best practices, as well as provide a solid foundation for decision-making and policy formulation. ^7^^,^^13^ Therefore, the aim of this study was to evaluate the scientific production in USR related to institutions in Latin America and the Caribbean, through bibliometric analysis of publications indexed in the Scopus database. It is expected to identify possible gaps and challenges in current scientific production, which will contribute to guiding future research and promoting academic collaboration in the region. ^5^^,^^14^

## Methods

### Study design

A bibliometric analysis was conducted on the scientific production of USR in institutions from Latin America and the Caribbean. Scientific articles indexed in the Scopus database were included from its inception until April 2023. Articles addressing USR and describing an experience conducted by a university in Latin America and the Caribbean were included, regardless of the study design.

### Database

The Scopus database was chosen for the present bibliometric analysis due to its breadth and coverage of scientific articles, as well as its availability of metadata useful for this type of analysis.

### Search strategy

The search strategy was conducted by one of the authors (GBQ) and included free terms used for searching the title, abstract, and keywords of the articles indexed in the Scopus database. The search formula is described in detail in a repository. ^15^ The search strategy was independently reviewed and evaluated by another author (DUP). The search was not restricted by year or language of publication.

### Data collection

Data for each research article found during the search were downloaded as a.csv file from Scopus and imported into the Rayyan website. Two authors (DUP and GBQ) reviewed the title and abstract of each article to assess compliance with the eligibility criteria.

### Bibliometric analysis

The Bibliometrix package in the R Studio statistical program was used for the analysis. One author (GBQ) manually standardized the names and affiliations of the authors found. The characteristics of the included articles were described, including the number of articles per author, average number of authors per article, average number of citations per article, and the number of documents with one or more author. Additionally, useful indicators for bibliometric evaluation, such as the authorship rate (ratio between the total number of articles and the total number of authors), co-authorship rate (average number of co-authors per article), and collaboration rate (ratio between the total number of authors of articles with multiple authors and the total number of articles with multiple authors) were described.

The VOSviewer software was used to construct and observe bibliometric networks based on bibliographic coupling analysis, using information on the countries of institutions with documents on USR and the co-occurrence of terms in titles and abstracts. No threshold was set for the inclusion of terms for the analysis of term co-occurrence. For this analysis, terms related to USR were excluded.

### Ethical considerations

Data from documents published in journals indexed in the Scopus bibliographic database, which do not include confidential data of human subjects, were analyzed. Therefore, ethical committee approval was not required for this study.

## Results

**Figure 1.**
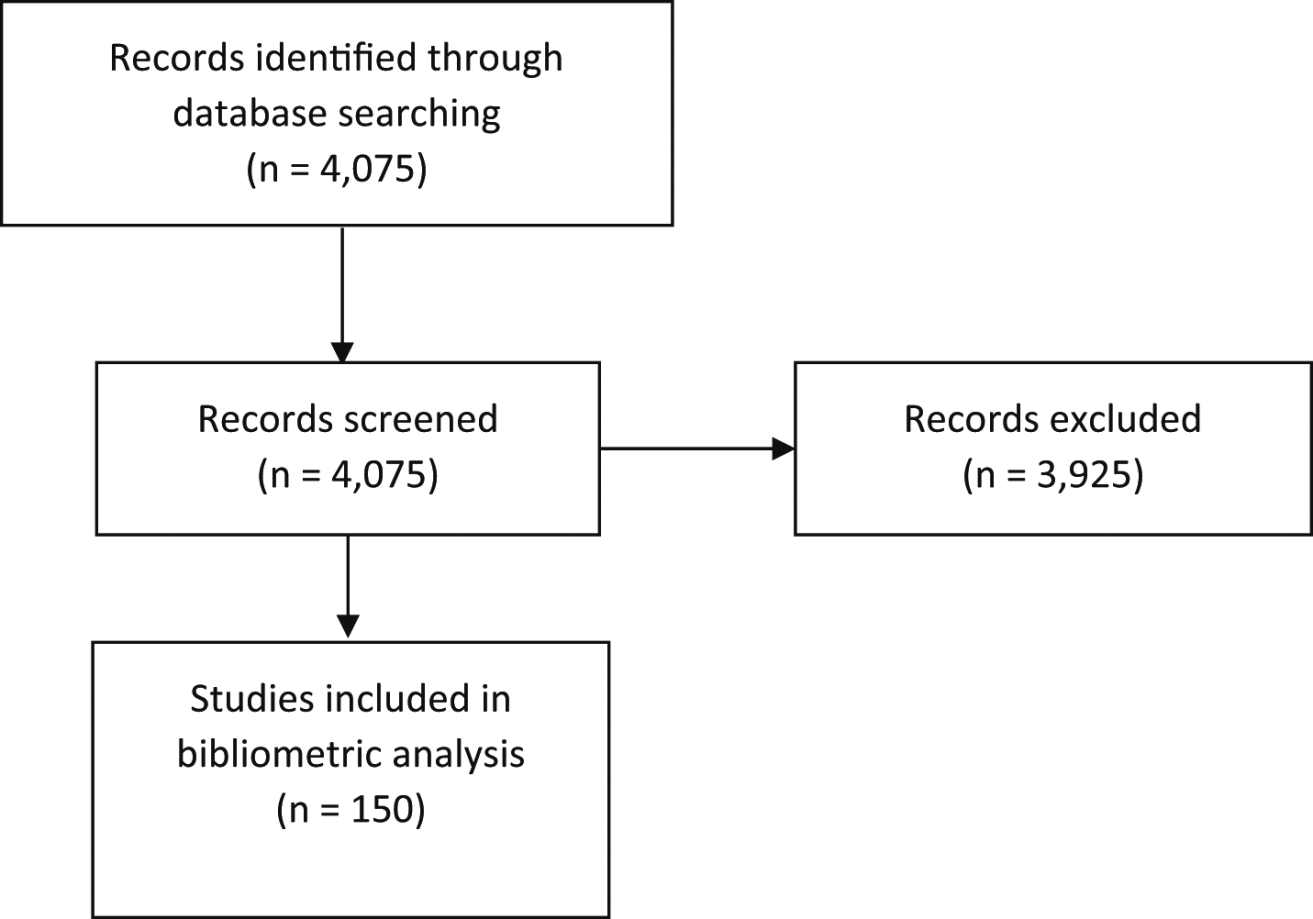
Flowchart of selection.

**Figure 2.**
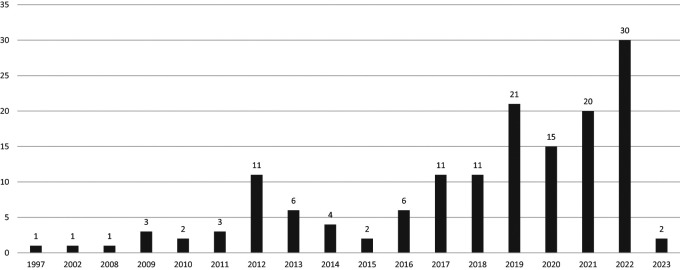
Scientific production in Scopus during the study period.

A total of 4,075 records were retrieved from the SCOPUS bibliographic database. After excluding documents that did not meet the eligibility criteria, 150 scientific articles were included ( Figure 1). We included articles published between 1997 and 2023 ( Figure 2). An increase in the number of scientific articles on USR was identified, with an average annual growth rate of 2.7%. The year 2022 had the highest number of published documents.

Regarding the authors of the documents, a total of 439 authors with 469 mentions were identified. On average, there were 0.3 articles per author, a co-authorship index per document of 3.13, and 18 articles had a single author. The percentage of international collaborations was 30.7%. The authors Severino-González P (12 articles) and Sarmiento-Peralta G (4 articles) had the highest number of scientific publications ( Table 1).

**Table 1.** Authors with the highest scientific production in Scopus related to university social responsibility in Latin America and the Caribbean.

Author	Number of publications
Severino-González P	12
Sarmiento-Peralta G	4
Rubio-Rodríguez Ga	3
Acuña-Moraga O	2
Calderón AI	2
Chalco KYM	2
Del Castillo CAS	2
Delgado FAD	2
Delgado MFF	2
García Martínez J	2
Machaca ESM	2
Martí-Noguera JJ	2
Quezada RG	2
Quintanilla KPB	2
Ramos Parra C	2
Romero-Argueta J	2
Vallaeys F	2
Acosta YC	1
Ahumada CA	1
Alberto RAG	1

**Figure 3.**
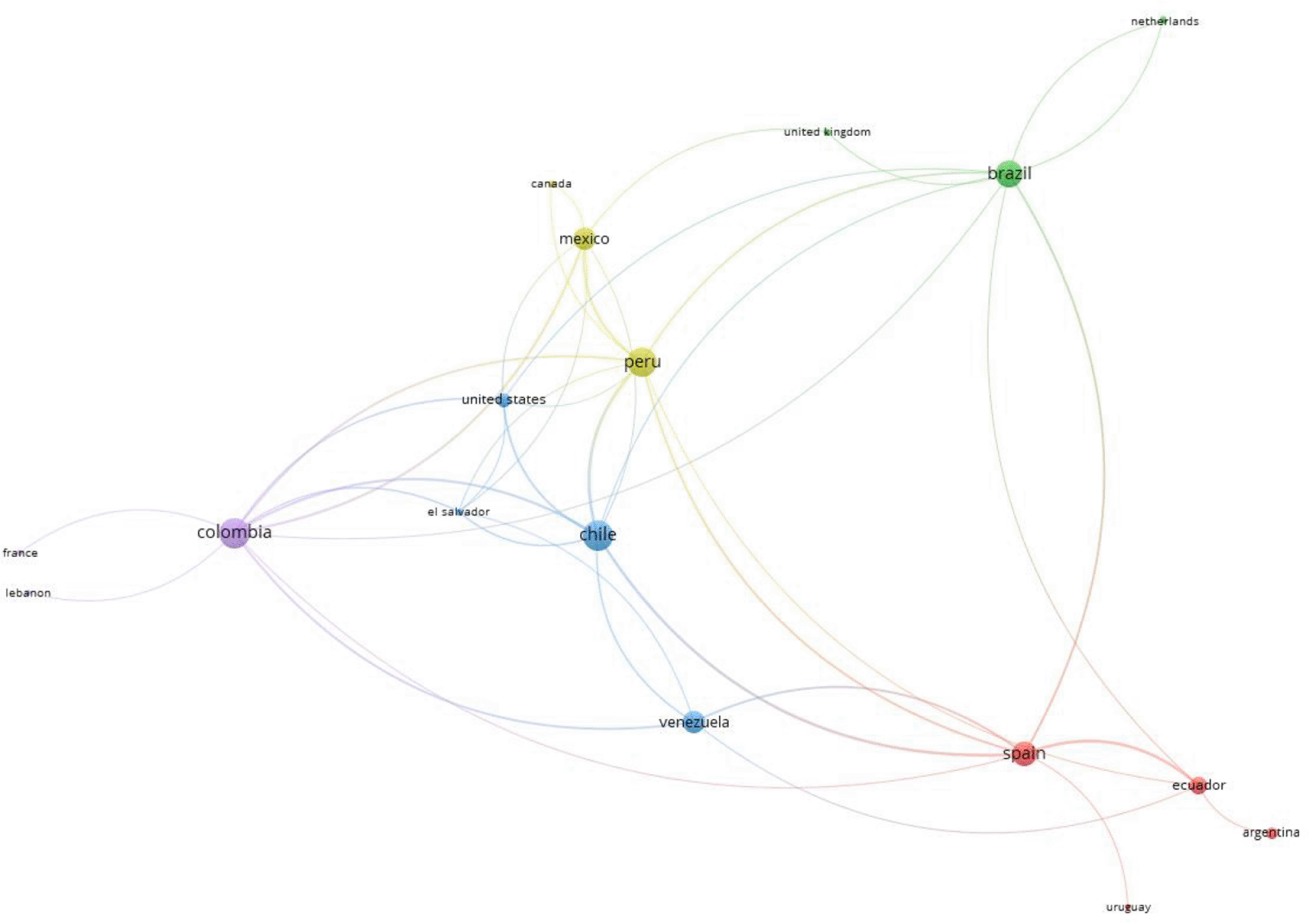
Collaboration network among countries on scientific production related to university social responsibility in Latin America and the Caribbean.

In terms of the country of origin of the institutional affiliation of the authors of the articles on USR, Brazil had the highest proportion of publications (26.4%), followed by Chile (17%) and Colombia (13.2%). Brazil had the highest number of documents as the sole country of institutional affiliations of the authors. Additionally, Brazil topped the list of countries with the highest number of citations and average citations per article, with 58 and 4.1, respectively, followed by Chile (49 and 5.4, respectively) and Colombia (45 and 6.4, respectively) ( Table 2). These three countries (Brazil, Chile, and Colombia), along with Peru, were of note in international network collaboration in publications on USR ( Figure 3).

**Table 2.** Countries with the highest production, number of citations, and average citations per article in Scopus related to university social responsibility in Latin America and the Caribbean.

Country	Total citations	Average citations per article
Brazil	58	4.1
Chile	49	5.4
Colombia	45	6.4
Spain	42	8.4
Lebanon	32	32
Venezuela	12	2.4
Mexico	7	1.4
USA	6	6
Ecuador	3	1
Argentina	2	2
Canada	2	2
Poland	1	1

Regarding citations, the articles had an average of 4.6 citations. The articles by Sánchez-Hernández MI (2016) and Vallaeys F (2019) had the highest number of citations (42 and 36, with an average citation rate of 5.3 and 7.2 per year, respectively). *Opción* and *Revista de Ciencias Sociales* were the journals in which the highest number of articles were published, with 13 each ( Table 3).

**Table 3.** Journals with the highest number of publications in Scopus related to university social responsibility in Latin America and the Caribbean.

Journal	Number of articles
Opción	13
Revista de Ciencias Sociales	13
Revista Venezolana de Gerencia	11
Espacios	8
Formación Universitaria	7
Universidad y Sociedad	5
Interciencia	3
International Journal of Educational Management	3
Revista Iberoamericana de Educación Superior	3
Sustainability (Switzerland)	3
World Sustainability Series	3
International Journal of Management in Education	2
Proceedings - JICV 2022: 12th International Conference on Virtual Campus	2
Proceedings of the LACCEI International Multi-Conference for Engineering Education and Technology	2
Revista Brasileira de Educacao	2
Revista de la Educacion Superior	2
Social Responsibility Journal	2
2019 IEEE Global Humanitarian Technology Conference GHTC 2019	1
3rd International Conference of the Portuguese Society for Engineering Education CISPEE 2018	1
American Behavioral Scientist	1

**Figure 4.**
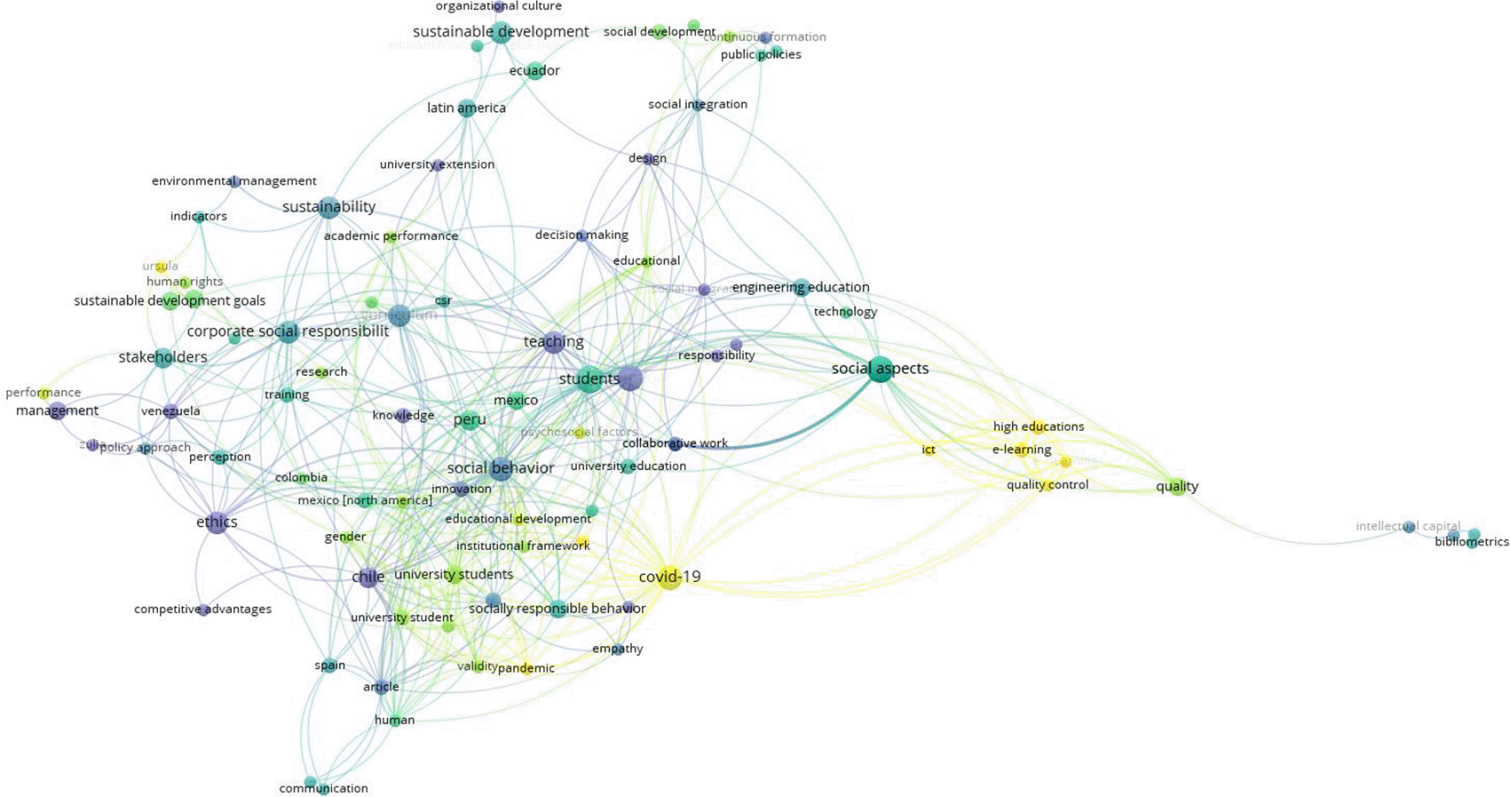
Analysis of term co-occurrence in titles and abstracts (overlap visualization) and its temporal evolution using VOSviewer software in relation to scientific publications in Scopus related to university social responsibility in Latin America and the Caribbean.

In the co-occurrence analysis of terms ( Figure 4), it was identified that in the early years, publications on USR referred to terms such as ethics, knowledge, and teaching. In more recent years, the use of terms related to e-learning, information and communication technologies, virtual education, COVID-19, sustainable development goals, and URSULA (initiative on USR in Latin American and Caribbean institutions) became evident.

## Discussion

In this study, we evaluated and characterized the scientific production related to USR by institutions in Latin America and the Caribbean using the Scopus database. We found an increase in scientific production over the past ten years, reaching a peak in 2022. According to the co-occurrence analysis of terms, recent documents on USR encompass topics related to the use of information and communication technologies, COVID-19, and sustainable development.

The observed increase in scientific production on USR by institutions in Latin America and the Caribbean could be attributed to a growing social awareness and commitment, manifested through increased investment and recognition by higher education institutions in social development. Additionally, due to significant social inequalities and challenges in areas such as poverty, education, health, and the environment, institutions in the region may show a greater interest in USR. ^16^^–^^18^ Promoting research in this region could be facilitated by establishing close and collaborative partnerships between communities and universities. This entails working together to identify and address social issues and promote sustainable development. ^6^ Furthermore, we identified peaks in scientific production between 2019 and 2022, which could be attributed to an upsurge in scientific output related to the COVID-19 pandemic. ^19^^–^^21^ This is evident in the co-occurrence analysis of terms, where the term "COVID-19" is of note as one of the most frequently mentioned.

There was a predominance of countries such as Brazil, Chile, and Colombia in the publication of documents on USR by institutions in Latin America and the Caribbean. This finding aligns with the scientific production of these countries, which lead in research output in the region. ^22^^–^^24^ It is worth noting that both the countries with the highest and lowest contributions to scientific production are of middle and high income, indicating no significant difference between income level and scientific contribution on USR. These results contrast with findings from other studies that have examined this potential relationship between income and scientific production. ^25^ Therefore, it is possible that country-level promotion and initiatives explain this finding in countries with higher scientific production.

The authors with the highest scientific production did not exceed 13 scientific articles in Scopus, with researchers affiliated with institutions in Chile, Peru, and Colombia being of note, respectively. These countries, along with Brazil, demonstrate clear regional collaboration as well as collaboration with countries with advanced scientific development, as shown in the collaborative network graph. In various fields of knowledge, north-south collaboration is described as part of the early research development process in countries with lower scientific development. ^26^^,^^27^ This influence may be observed in the development of research on USR in the Latin American and Caribbean region. On the other hand, the gap between the top-producing author and the second highest is eight articles, which reinforces the idea that USR is a young and growing field.

The scientific journal with the highest number of articles published was *Opción*, followed by the *Revista de Ciencias Sociales* and the *Revista Venezolana de Gerencia.* These journals publish papers related to social sciences, humanities, and education. This is consistent with a previous study, ^28^ which explains that USR primarily falls within the field of social sciences, education, and humanities. Therefore, documents on USR from institutions in Latin America and the Caribbean would be of interest to these journals and their readers. All three journals are Venezuelan and have been indexed in Scopus for no more than 15 years. This aligns with the years when an increase in scientific production related to USR in Latin American institutions was observed.

The co-occurrence analysis of terms indicates that documents on USR from Latin American and Caribbean institutions have shifted from general aspects, such as ethics and education, to focusing on current challenges, such as the context of the COVID-19 pandemic, virtual education, and the use of information and communication technologies. As described in other fields, it is expected that over the years publications on a topic, such as USR, would shift focus from generalities to addressing its current applicability and challenges. Among the recently used terms is URSULA (Latin American Union for University Social Responsibility), which seeks to provide innovative proposals to improve the social and environmental role of universities through dialogue among different stakeholders, such as civil society, governments, scientists, and businesses. ^29^ In recent years, some Latin American countries have worked on implementing policies and programs that promote USR. ^6^^,^^14^^,^^30^ These initiatives may include incentives, funding, or specific requirements for academic institutions to conduct research and projects aimed at social well-being. This situation, combined with increased access to resources and technology in academic institutions, would generate greater opportunities for scientific research and the dissemination of its results, which could also explain the increase in scientific production on USR in the region.

To the best of our knowledge, this bibliometric analysis is the first to evaluate the scientific production on USR associated with institutions in Latin America. The results are valuable for identifying the current status and growth of this area. Additionally, we can identify authors who work in this field and the journals that frequently publish related articles. Among the potential limitations of this research, it should be mentioned that articles published in databases other than Scopus were not included, which means that some documents on USR from institutions in Latin America and the Caribbean may not have been considered. However, we believe that using Scopus, a reliable and widely employed source for bibliometric studies, ensures that the results obtained are from documents published in journals with quality criteria, such as peer review, and the requirements demanded by the bibliographic database for the inclusion of indexed journals, that is, the documents retrieved through the search strategy in this study. In this bibliometric analysis, a review of the title and abstract of the documents was conducted for their inclusion in the analysis, which strengthens the study as it ensured the inclusion of only documents on USR.

## Conclusions

In conclusion, there is an increase in scientific production on USR by researchers from institutions in Latin America and the Caribbean. The countries leading the research in the region also show leadership in documents on USR. In recent years, the focus of documents on USR has been on COVID-19, virtual education, and sustainable development. Quantifying the scientific production on USR in Latin America and the Caribbean provides a baseline for future research in the field.

## Data Availability

Figshare: Search strategy. DOI: https://doi.org/10.6084/m9.figshare.24179106. ^15^ This project contains the following underlying data:
• An.docx file containing Scopus search strategy used to perform this bibliometric analysis. Figshare: Database containing the articles included in the analysis. DOI: https://doi.org/10.6084/m9.figshare.24069399. ^31^ This project contains the following underlying data:
• An.xls file containing the database of articles included in the bibliometric analysis is available. Data are available under the terms of the Creative Commons Zero “No rights reserved” data waiver (CC BY 4.0 Public domain dedication).
