# Supplementary material for: Bibliometric analysis of scientific production on university social responsibility in Latin America and the Caribbean
Source: F1000Res. 2024 Jul 18;12:1340. Originally published 2023 Oct 16. [Version 2] doi: 10.12688/f1000research.141987.2 (PMC11289591; doi:10.12688/f1000research.141987.2)
Supplement: Peer review history

# Reviewer report: Reviewer response for version 2

**DOI:** 10.5256/f1000research.168804.r304719

**Published:** 2024-07-30

**Authors:** Maria Jesus Martinez Usarralde, Nicolás Ponce Diaz

**Affiliations:** 1 Department of Comparative Education and History of Education, University of Valencia, Valencia, Spain; 2 Facultad de Educación, University of Antofagasta, Antofagasta, Antofagasta Region, Chile

The revisions are appropriate for me.

Is the work clearly and accurately presented and does it cite the current literature?

Yes

If applicable, is the statistical analysis and its interpretation appropriate?

I cannot comment. A qualified statistician is required.

Are all the source data underlying the results available to ensure full reproducibility?

Partly

Is the study design appropriate and is the work technically sound?

Partly

Are the conclusions drawn adequately supported by the results?

No

Are sufficient details of methods and analysis provided to allow replication by others?

Partly

Reviewer Expertise:

Comparative Education. International Education. Teaching Education. Service Learning.

We confirm that we have read this submission and believe that we have an appropriate level of expertise to confirm that it is of an acceptable scientific standard.

# Reviewer report: Reviewer response for version 2

**DOI:** 10.5256/f1000research.168804.r304720

**Published:** 2024-07-26

**Authors:** Mikel Perez-Gutierrez

**Affiliations:** 1 Universidad de Cantabria, Santander, Cantabria, Spain

The quality of the article was improved, clarifying the methodology, results, discussion and conclusions sections.

 Minor errors were detected. In Table 2, Brazil is not top regarding average citations per article (Spain obtained 8.4) so the sentence should be amended. In the conclusions section, URS was used instead of USR. This error should be amended.

Is the work clearly and accurately presented and does it cite the current literature?

Yes

If applicable, is the statistical analysis and its interpretation appropriate?

Yes

Are all the source data underlying the results available to ensure full reproducibility?

Yes

Is the study design appropriate and is the work technically sound?

Yes

Are the conclusions drawn adequately supported by the results?

Partly

Are sufficient details of methods and analysis provided to allow replication by others?

Partly

Reviewer Expertise:

Bibliometrics, Sport Sciences, Physical Education

I confirm that I have read this submission and believe that I have an appropriate level of expertise to confirm that it is of an acceptable scientific standard.

# Reviewer report: Reviewer response for version 1

**DOI:** 10.5256/f1000research.155478.r225262

**Published:** 2024-02-14

**Authors:** Mikel Perez-Gutierrez

**Affiliations:** 1 Universidad de Cantabria, Santander, Cantabria, Spain

The manuscript carried out a bibliometric analysis of the scientific production indexed in Scopus focused on University Social Responsibility by Latin America and Caribbean institutions. The manuscript offers an overview of the main bibliometric indicators for characterising this scientific output. However, some deficiencies should be amended for improving the quality and reproducibility of the research (methodology section) as well as the understanding of the obtained results (results, discussion and conclusions sections). These suggestions are the following:

- Page 3: For improving the understanding of the manuscript and its object of study, Latin America and the Caribbean countries should be defined and justified. For that purpose, CELAC (Comunidad de Estados Latinoamericanos y Caribeños) could be used  for defining the countries considered under the term "Latin America and the Caribbe". For citation, these pages should be consulted and cited: http://s017.sela.org/celac/quienes-somos/que-es-la-celac/ or https://es.wikipedia.org/wiki/Comunidad_de_Estados_Latinoamericanos_y_Caribe%C3%B1os),
- Page 3: A justification of the database chosen for data retrieval should be included. The work carried out by Gusenbauer and Haddaway (2020) analysed the coverage and breadth of several databases, concluding Scopus is a principal database  and justifying its use in the present manuscript. (Gusenbauer et al) ^1^
- Page 3: All bibliometric indicators included in the results section should be explained. Total citations and the average citations per article by each country are included in Table 2. Table 3 presents the number of articles published in the most important journals. These indicators should be mentioned here.
- Page 3: According to the results presented in Figure 4, an explanation about the analysis applied for the temporal evolution of the term co-occurrence and the presentation of results (temporal evolution by means of colours?) should be included. How this temporal evolution is presented in Figure 4?
- Page 4: The difference between "author" and "mention" should be explained in the methodology section. Are "mentions" referring to "signatures"?
- Page 4: Regarding the obtained results presented in Table 2, the highest average of citations per article was obtained by Lebanon. If authors are referring to the Latin America and Caribbean countries with the highest average citations per article, Colombia achieved the highest (8.4), followed by Chile (5.4) and Brazil (4.1). The sentence should be amended for clarifying this information.
- Page 5: The title of Table 1 (Authors with the highest scientific production) is not representing the results included since there are three authors with one paper, but there are more authors with only one paper. The title of the table and the results included should be amended. Firstly, authors with 2 or more papers should be only included in Table 1. Thus, the title of Table 1 should be the following: "Authors with two or more articles indexed in Scopus..."
- Page 5: Table 2 should also include the amount of papers by country for comparing their productivity. Moreover, in the results section the percentage of publications per country is described (Brazil 26.4%, Chile 17% and Colombia 13.2%).
- Page 6: As it was mentioned in Table 1, the title of Table 3 (Journals with the highest scientific production) is not representing the results included since there are three journals with one paper, but it seems there are more journals with only one paper. The title of the table and the results included should be amended. Firstly, journals with 3 or more papers should be only included in Table 3. Thus, the title of Table 3 should be the following: "Journals with three or more publications..."
- Page 7: The results obtained in the present manuscript should be compared with those obtained by Duque and Cervantes-Cervantes (2019) since their topic is the same. Specifically, results could be compared according to total number of articles focused on University Social Responsibility, distribution of articles by country, distribution of articles by year, most productive authors and most important journals for disseminating USR scientific output. This comparison is relevant for determining the contribution of Latin America and Caribbean countries, institutions or authors to the worldwide scientific output in this topic.
- Page 8: "To the best of our knowledge, this bibliometric analysis is the first to evaluate the scientific production on USR associated with institutions in Latin America". According to the bibliometric analysis developed, the present manuscript is evaluating the scientific production on USR published by Latin America and Caribbean institutions. The sentence should be amended.
- Page 8: The conclusion section should be strengthened. An explanation about the causes and consequences of this analysis should be included. Some questions to answer are: What is the relevance of Latin America and Caribbean institutions regarding USR scientific output? Who is the most important author/country? Is collaboration among authors, institutions or countries essential for developing research on USR? What are the strengths and weaknesses of this filed of knowledge? What knowledge gaps need to be filled? To which countries should investment in research be directed?

Is the work clearly and accurately presented and does it cite the current literature?

Yes

If applicable, is the statistical analysis and its interpretation appropriate?

Yes

Are all the source data underlying the results available to ensure full reproducibility?

Yes

Is the study design appropriate and is the work technically sound?

Yes

Are the conclusions drawn adequately supported by the results?

Partly

Are sufficient details of methods and analysis provided to allow replication by others?

Partly

Reviewer Expertise:

Bibliometrics, Sport Sciences, Physical Education

I confirm that I have read this submission and believe that I have an appropriate level of expertise to confirm that it is of an acceptable scientific standard, however I have significant reservations, as outlined above.

## References

[1] : Which academic search systems are suitable for systematic reviews or meta-analyses? Evaluating retrieval qualities of Google Scholar, PubMed, and 26 other resources. *Res Synth Methods* .2020;11(2) : 10.1002/jrsm.1378 181-217 10.1002/jrsm.1378 31614060 PMC7079055

# Response

**Published:** 2024-07-03

**Authors:** Diego Urrunaga-Pastor

**Affiliations:** USIL, Peru

Dear Reviewer,

 We appreciate your review of our manuscript and the comments you provided. Below, we have addressed each of your observations in detail:

 Comment 1: Page 3: For improving the understanding of the manuscript and its object of study, Latin America and the Caribbean countries should be defined and justified. For that purpose, CELAC (Comunidad de Estados Latinoamericanos y Caribeños) could be used for defining the countries considered under the term "Latin America and the Caribbean". For citation, these pages should be consulted and cited: [http://s017.sela.org/celac/quienes-somos/que-es-la-celac/](http://s017.sela.org/celac/quienes-somos/que-es-la-celac/) or [https://es.wikipedia.org/wiki/Comunidad_de_Estados_Latinoamericanos_y_Caribe%C3%B1os](https://es.wikipedia.org/wiki/Comunidad_de_Estados_Latinoamericanos_y_Caribe%C3%B1os).

 Response: We appreciate your comment. All the document sections were reviewed and improved. Since the provided reference by the reviewer was not available, the Latin America & Caribbean countries list by United Nations was used.

 Comment 2: Page 3: A justification of the database chosen for data retrieval should be included. The work carried out by Gusenbauer and Haddaway (2020) analyzed the coverage and breadth of several databases, concluding Scopus is a principal database and justifying its use in the present manuscript.

 Response: We appreciate your comment. We cited documents in the methods to justify the database used (Scopus).

 Comment 3: Page 3: All bibliometric indicators included in the results section should be explained. Total citations and the average citations per article by each country are included in Table 2. Table 3 presents the number of articles published in the most important journals. These indicators should be mentioned here.

 Response: We appreciate your comment. Regarding the clarification of all bibliometric indicators, there are stated in the methods: “Additionally, useful indicators for bibliometric evaluation, such as the authorship rate (ratio between the total number of articles and the total number of authors), co-authorship rate (average number of co-authors per article), and collaboration rate (ratio between the total number of authors of articles with multiple authors and the total number of articles with multiple authors) were described.” The information about the results presented in Tables 2 and 3 was added to the methods.

 Comment 4: Page 3: According to the results presented in Figure 4, an explanation about the analysis applied for the temporal evolution of the term co-occurrence and the presentation of results (temporal evolution by means of colours?) should be included. How is this temporal evolution presented in Figure 4?

 Response: We appreciate your comment. In methods, information was added to clarify. Additionally, the figure 2 was reuploaded since in the reviewed manuscript, it seems that the legend (colors according to the timeline in years) was not presented. 

 Comment 5: Page 4: The difference between "author" and "mention" should be explained in the methodology section. Are "mentions" referring to "signatures"?

 Response: We appreciate your comment. This information was confusing. We improve the redaction.

 Comment 6: Page 4: Regarding the obtained results presented in Table 2, the highest average of citations per article was obtained by Lebanon. If authors are referring to the Latin America and Caribbean countries with the highest average citations per article, Colombia achieved the highest (8.4), followed by Chile (5.4) and Brazil (4.1). The sentence should be amended for clarifying this information.

 Response: We appreciate your comment. This table was improved, and the information reported about this table.

 Comment 7: Page 5: The title of Table 1 (Authors with the highest scientific production) is not representing the results included since there are three authors with one paper, but there are more authors with only one paper. The title of the table and the results included should be amended. Firstly, authors with 2 or more papers should be only included in Table 1. Thus, the title of Table 1 should be the following: "Authors with two or more articles indexed in Scopus..."

 Response: We appreciate your comment. Table 1 was updated with the suggestions.

 Comment 8: Page 5: Table 2 should also include the amount of papers by country for comparing their productivity. Moreover, in the results section, the percentage of publications per country is described (Brazil 26.4%, Chile 17% and Colombia 13.2%).

 Response: We appreciate your comment. This table was improved, and the information reported about this table.

 Comment 9: Page 6: As it was mentioned in Table 1, the title of Table 3 (Journals with the highest scientific production) is not representing the results included since there are three journals with one paper, but it seems there are more journals with only one paper. The title of the table and the results included should be amended. Firstly, journals with 3 or more papers should be only included in Table 3. Thus, the title of Table 3 should be the following: "Journals with three or more publications..."

 Response: We appreciate your comment. The table was updated with the suggestion.

 Comment 10: Page 7: The results obtained in the present manuscript should be compared with those obtained by Duque and Cervantes-Cervantes (2019) since their topic is the same. Specifically, results could be compared according to total number of articles focused on University Social Responsibility, distribution of articles by country, distribution of articles by year, most productive authors and most important journals for disseminating USR scientific output. This comparison is relevant for determining the contribution of Latin America and Caribbean countries, institutions or authors to the worldwide scientific output in this topic.

 Response: We appreciate your comment. We added the recommended document in the discussion of the results.

 Comment 11: Page 8: "To the best of our knowledge, this bibliometric analysis is the first to evaluate the scientific production on USR associated with institutions in Latin America". According to the bibliometric analysis developed, the present manuscript is evaluating the scientific production on USR published by Latin America and Caribbean institutions. The sentence should be amended.

 Response: We appreciate your comment. The sentence was amended.

 Comment 12: Page 8: The conclusion section should be strengthened. An explanation about the causes and consequences of this analysis should be included. Some questions to answer are: What is the relevance of Latin America and Caribbean institutions regarding USR scientific output? Who is the most important author/country? Is collaboration among authors, institutions or countries essential for developing research on USR? What are the strengths and weaknesses of this field of knowledge? What knowledge gaps need to be filled? To which countries should investment in research be directed?

 Response: We appreciate your comment. The conclusion was improved

# Reviewer report: Reviewer response for version 1

**DOI:** 10.5256/f1000research.155478.r217335

**Published:** 2023-11-13

**Authors:** Maria Jesus Martinez Usarralde, Nicolás Ponce Diaz

**Affiliations:** 1 Department of Comparative Education and History of Education, University of Valencia, Valencia, Spain; 2 Facultad de Educación, University of Antofagasta, Antofagasta, Antofagasta Region, Chile

The article needs some improvement:

- The introduction is excessively brief, as is the conclusion.
- In the "search strategy" section, they point out that they have restricted language and year, but the Vosviewer programme only codes articles in English.
- "There are no significant differences between high and low income countries". This is not being evidenced and is based on a study that has nothing to do with the research field.
- Paragraph on co-occurrence: the desire expressed by the authors cannot be derived from a co-occurrence analysis.
- "The bibliometric analysis is the first to evaluate the scientific production of USR associated with Latin American institutions": the analysis referred to in the article is not observed in this article, in the sense that the data do not reflect the scientific production of USR in Latin American institutions.

Is the work clearly and accurately presented and does it cite the current literature?

Yes

If applicable, is the statistical analysis and its interpretation appropriate?

I cannot comment. A qualified statistician is required.

Are all the source data underlying the results available to ensure full reproducibility?

Partly

Is the study design appropriate and is the work technically sound?

Partly

Are the conclusions drawn adequately supported by the results?

No

Are sufficient details of methods and analysis provided to allow replication by others?

Partly

Reviewer Expertise:

Comparative Education. International Education. Teaching Education. Service Learning.

We confirm that we have read this submission and believe that we have an appropriate level of expertise to confirm that it is of an acceptable scientific standard, however we have significant reservations, as outlined above.

# Response

**Published:** 2024-07-03

**Authors:** Diego Urrunaga-Pastor

**Affiliations:** USIL, Peru

Dear Reviewers,

 We appreciate your review of our manuscript and the comments you provided. Below, we have addressed each of your observations in detail:

 Comment 1: The introduction is excessively brief, as is the conclusion.

 Response:

 We appreciate your comment. Relevant Information was added to the manuscript regarding bibliometric analysis.

 Comment 2: In the "search strategy" section, they point out that they have restricted language and year, but the Vosviewer programme only codes articles in English.

 Response:

 We appreciate your comment. The document states, “The search was not restricted by year or language of publication.” No change was conducted.

 Comment 3: "There are no significant differences between high- and low-income countries". This is not being evidenced and is based on a study that has nothing to do with the research field.

 Response:

 We appreciate your comment. The redaction was improved. The writing was improved according to the comment.

 Comment 4: Paragraph on co-occurrence: the desire expressed by the authors cannot be derived from a co-occurrence analysis.

 Response:

 Thank you for the recommendation. Regarding the co-occurrence overlay visualization, the attribute average publication year of the document in which a keyword or a term occurs was used in the document. As it is described in the VOSViewer manual about this attribute, “The average publication year of the documents in which a keyword or a term occurs or the average publication year of the documents published by a source, an author, an organization, or a country.” Hence, the interpretation of this visualization is appropriate for discussion. Information was added in methods to clarify.

 Comment 5: "The bibliometric analysis is the first to evaluate the scientific production of USR associated with Latin American institutions": the analysis referred to in the article is not observed in this article, in the sense that the data do not reflect the scientific production of USR in Latin American institutions.

 Response:

 We appreciate your comment. The expression “associated with” was replaced with “from” to be more precise as it is stated in the reviewer comment.
